# Gastrointestinal delivery of bitter hop extract reduces appetite and food cravings in healthy adult women undergoing acute fasting

**DOI:** 10.1016/j.obpill.2024.100117

**Published:** 2024-06-20

**Authors:** Edward Walker, Kim Lo, Pramod Gopal

**Affiliations:** a The New Zealand Institute for Plant and Food Research Limited, Mt Albert Research Centre, 120 Mt Albert Road, Mt Albert, Auckland, 1025, New Zealand; b The New Zealand Institute for Plant and Food Research Limited, Palmerston North, New Zealand

**Keywords:** Fasting, Bitter taste receptors, Appetite, Satiety, Water fasting, Dietary supplement

## Abstract

**Background:**

Dietary restrictions or reductions such as fasting for weight loss are often difficult to adhere to due to increased appetite and food cravings. Recently, gastrointestinal delivery of bitter hops has been shown to be effective at reducing appetite in men. Our aim was to determine the effect of a bitter hop extract on appetite and cravings in women, using a 24 h, water-only fast.

**Methods:**

This was a randomized, double-blind, cross-over treatment study. Thirty adult women were recruited and required to fast for 24 h from 1800 h to 1800 h on three occasions and given an *ad libitum* meal to break each fast. Treatments of either a placebo or one of two doses (high dose; HD: 250 mg or low dose; LD: 125 mg) of a bitter hop-based appetite suppressant (Amarasate®) were given twice per day at 16 and 20 h into the fast.

**Results:**

The HD and LD treatment groups exhibited a significant (*p* < 0.05) reduction in appetite and cravings for food when compared to the placebo control. Two participants reported loose stools and one reported heartburn while on the HD treatment, and one participant reported loose stools while on the LD treatment.

**Conclusion:**

These data suggest that appetite suppressant co-therapy may be useful in reducing hunger during fasting in women and shows that gastrointestinal delivery of bitter compounds may also be an effective method of reducing cravings for food.

This trial received ethical approval from the Northen B New Zealand Human Disability and Ethics committee (Northern B Health and Disability Ethics Committee (2022 EXP 10995) and was prospectively registered with the Australian New Zealand Clinical Trial Registry (ACTRN12622000107729).

## Highlights

• Gastrointestinal (GI) targeted hop extract reduced hunger and food cravings in females undergoing an acute 24 h fast.
• Post fast *ad libitum* energy intake was reduced by up to 14 %.
• Pre-meal craving for savory food and post-meal craving for sugary foods was reduced by hop extract intervention.
• Hop extract treatment improved markers of compliance during the fast and may assist those undergoing fasting type diets.

## Graphical abstract

**Figure:**
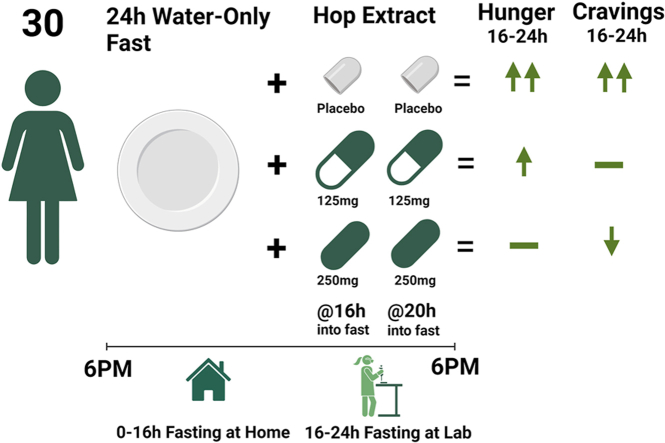

## 1 Introduction

Bitter tasting plants have a long history of use as traditional, health-giving foods and medicine, with recent interest in their application as appetite suppressants. The use of bitter foods to suppress appetite and food cravings is reported in several cultures throughout history with, for example, bitter herbs such as guggul being used in Ayurvedic medicine to reduce food intake, stimulate weight loss and suppress appetite [1,2]. Interestingly, two examples of traditional bitter appetite suppressants have recently been rediscovered and considered for commercial development. *Lathyrus linifolius* (heath pea) is reported to have been used in the highlands of Scotland during medieval times to reduce hunger during times of food scarcity and is reported to have been used in the court of King Charles II of England as a weight-loss treatment. *Hoodia gordonii*, a bitter succulent plant native to Africa was used by the San people of the Kalahari Desert to reduce appetite during long hunting trips [3].

The effectiveness of directed GI bitterness for appetite regulation was assessed in a recent 2021 meta-analysis [4]. Klaassen et al. determined that pre-meal treatment with bitter tastants significantly reduce energy intake, concluding that “Bitter stimuli are most potent to influence eating behavior”, and suggested their mechanism of action was likely via the stimulation of appetite-suppressing gut peptide hormones released from enteroendocrine cells [4]. Although bioactive, most of the bitter compounds evaluated in this meta-analysis were either non-dietary, or pharmaceutical compounds, or unsuitable for development into a consumer product. The search for a commonly consumed bitter plant extract with a history of medicinal use that could be used for safe and effective appetite control led to *Humulus lupulus* (hops). Hops have been used medicinally since medieval times, including as an essential ingredient in beer brewing for well over 1000 years. Over this time, hops have been bred for increased bittering properties. The primary bittering components of hops are the alpha acids, with some additional bitterness being contributed by the beta acids in the hop [5]. These bioactive hop compounds exhibit limited bioavailability [6], persistent bitterness in epithelial sensory tissue [7], and high viscosity when in extract form. These properties are favorable for producing a potent and persistent bitterness at the gut surface when delivered directly to the GI tract in a highly concentrated capsule-based format.

We have previously shown that capsule-based GI delivery of an extract of bitter hop can reduce food intake, suppress appetite, and stimulate the release of appetite-suppressing gut hormones in healthy males [8,9]. To the best of our knowledge, however, no published work exists that assess the efficacy of bitter hops on appetite in females or on the effect of bitter hops on food cravings. Determining the efficacy of bitter hops in the regulation of food cravings and appetite measures in females is important, as the effect of GI bitterness on appetite may differ between males and females [10], and as food cravings are key determinants in diet compliance [11]. In this study we hypothesis that an extract of New Zealand hops (Amarasate®) will 1) Improve subjective measures of appetite and food craving during the 16–24 h period of a 24 h water-only fast in healthy, normal weight women, and 2) Reduced rebound eating immediately post fast.

## 2 Materials and methods

### 2.1 Participants

Thirty healthy women (eligibility: age 18–40 y, BMI 18.5–25 kg/m^2^) were recruited through poster and electronic advertisement in Auckland, New Zealand. The exclusion criteria were: current participation in a weight loss program or use of weight loss medication; diagnosed diseases of the GI tract; previous GI surgery; undergoing food restriction or any condition that may alter body composition within a short period of time; recent weight loss/gain (5 kg within the last 6 months); use of nonsteroidal anti-inflammatory drugs or current glucocorticoid use; history of ischemic gut, diabetes, celiac disease; history of alcohol or drug abuse; any known medical conditions/medications that may affect the gut or appetite; known allergy, intolerance, or sensitivity, to any ingredients in the study product; or inconsistent menstrual cycle. Participants were also required to be non-smokers, ascertained healthy by self-report and able to undertake a 24 h water-only fast by self-report. All subjects provided written informed consent prior to the appetite clinical trial and were allowed to withdraw at any time for any reason. Clinical research was approved by the Northern B Health and Disability Ethics Committee (2022 EXP 10995). All studies were carried out in accordance with the Declaration of Helsinki and the trial was registered at the Australian New Zealand Clinical Trials Registry (ACTRN12622000107729). The trial was conducted at The New Zealand Institute for Plant and Food Research Limited (PFR), Auckland, New Zealand, during 2022.

### 2.2 Study design, supplements and protocol

**Fig. 1:**
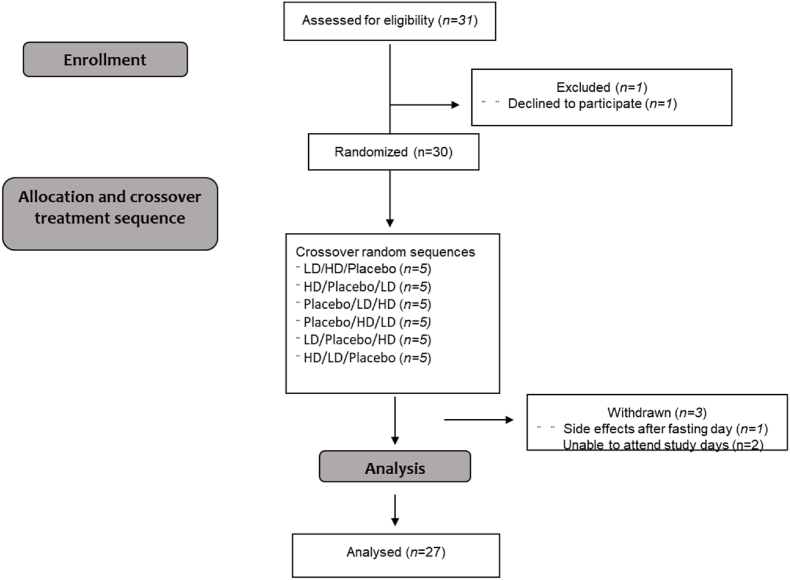
Consolidated Standards of Reporting Trials (CONSORT) flow diagram of the recruitment, enrollment, and random assignment process. HD (High Dose — 500 mg Amarasate®), LD (Low Dose — 250 mg Amarasate).

The study design was modified from a previous study examining the effect of the same hop extract on appetite measure in men [8]. Briefly, this was a randomized, double-blind, cross-over treatment study, using Visual Analogue Scales (VAS) to assess the effect of a hop extract on appetite and food craving during the last 8 h of a 24 h water-only fast. It involved two concentrations of the Amarasate hop flower extract suspended in a canola oil excipient and a placebo control, which were protected from gastric acid digestion by encapsulation using a hydroxypropyl methylcellulose (HPMC) DRcap capsule (DRcaps™, Capsugel, Morristown NJ, USA). The Amarasate hop extract was prepared using a food safe supercritical CO_2_ extraction process from a specific cultivar of *Humulus lupulus*. The intervention arms were two formulation matched dosages of the Amarasate extract, high dose (HD, 500 mg total) and low dose (LD, 250 mg total), and a placebo treatment. All treatment groups received two capsules, one given at 16 h (t = 0 min, 1000 h) and the second given 20 h (t = 240 min, 1400 h) into the 24 h fast, each containing half the total daily treatment dose. Treatment capsules were produced in a single batch and have previously been shown to be compositionally stable [8]. Participants were required to attend three study visits and were asked to refrain from excessive exercise, physical activity, and alcohol consumption for 24 h prior to the study day. Randomization was conducted using a Williams design balanced for order of presentation and carry-over effects. The Consolidated Standards of Reporting Trials (CONSORT) flow diagram is shown in Fig. 1.

**Fig. 2:**
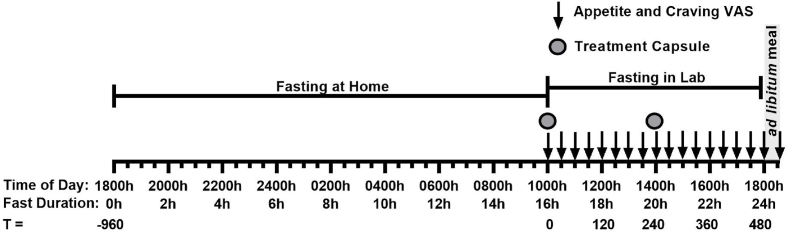
Study protocol on days 1–3. At 16 h into the 24 h fast (t = 0 min) participants recorded their baseline Visual Analogue Scale (VAS)-based appetite assessments and then consumed a delayed digestion capsule containing either a placebo, a low-dose Amarasate® hop extract or a high-dose Amarasate hop extract. Every 30 min, VAS-based appetite assessment questionnaires were taken from 16 h until 24 h into the 24 h fast. Then at 20 h into the 24 h fast (t = 240 min) a second treatment capsule matched to the first was given.

This trial included an *ad libitum* rice-based meal to end the 24 h fast (t = 480, 1800 h) and VAS assessments related to food cravings, as modifications from the original study design [8]. On the evening prior to commencement of the 24 h fast, participants were instructed to eat a typical, what they would normally eat, dinner until comfortably full immediately before 1800 h (t = −960 min) and then fast overnight with the consumption of water allowed. Participants arrived at the clinical facility at 0950 h (t = −10 min, 15 h 50 min ​into the fast) and were randomly assigned to one of three treatments after baseline measures were taken. Treatment capsules were given at 1000 h (t = 0 min, 16 h into fast) and at 1400 h (t = 240 min, 20 h into fast), presented in an opaque cup and consumed without participants’ handling the capsules. Appetite and food craving measures were taken at 30-min ​intervals from 1000 h (t = 0 min, 16 h into fast) by VAS (Fig. 2). When capsule administration and VAS assessment occurred at the same time, VAS were conducted immediately before capsule consumption. Water intake was recorded during the 16–24 h fasting period and participants were restricted from drinking immediately prior to and during VAS assessments. A rice-based meal was provided in excess at the end of the 24 h fast with participants being asked to eat until comfortably full. Participants were required to stay at the testing facility and remain sedentary until the study day was completed, and no sleeping was allowed (Fig. 2).

### 2.3 Appetite measures

Subjective measures of appetite (i.e., hunger, fullness, satisfaction, thoughts of food (TOF)), food-type cravings and physical comfort (e.g., nausea) were recorded from 16 h to 24 h of the fast and assessed by participants marking their appropriate subjective feelings on a 100 mm scale. These measures have previously been validated for appetite and food craving assessments, save for the bitter and spicy craving questions that are unique to this study [12]. The appetite-related VAS questions were as follows:

• “How hungry do you feel?” (‘I am not hungry at all’ to ‘I am as hungry as I have ever been’)
• “How full do you feel?” (‘I am not full at all’ to ‘I am totally full’)
• “How satisfied do you feel?” (‘I am completely empty’ to ‘I cannot eat another bite’)
• “How much do you think you can eat?” (‘nothing at all’ to ‘a large amount’).

The Food Craving-related VAS questions were as follows:

• “How much are you craving for food?” (‘not at all’ to ‘a great amount’)
• “How much are you craving for sugary foods?” (‘not at all’ to ‘a great amount’)
• “How much are you craving for salty foods?” (‘not at all’ to ‘a great amount’)
• “How much are you craving for fatty foods?” (‘not at all’ to ‘a great amount’)
• “How much are you craving for savory foods?” (‘not at all’ to ‘a great amount’)
• “How much are you craving for spicy foods?” (‘not at all’ to ‘a great amount’)
• “How much are you craving for bitter foods?” (‘not at all’ to ‘a great amount’).

VAS were measured immediately prior to, and then at 30, 60, 90, 120, 150, 180, 210, 240, 270, 300, 330, 390, 420, 450 and 480 min, after the first treatment capsule, then at 510 min, after the *ad libitum* meal. The primary outcomes of this trial were subjective measures of hunger, fullness, and satisfaction, and this study was powered to detect a 10 mm (10 %) change in VAS relative to the placebo as significantly different, as this is considered a meaningful behavioral change [13].

### 2.4 Fast breaking *ad libitum* meal

A fast-breaking, rice-based *ad libitum* meal was presented in excess in sensory booths to participants at 1800 h. The meal consisted of rice with non-meat additives, and had a macronutrient makeup of 4 g protein, 3 g of fat, and 29 g of carbohydrate (±1 g) per 100 g. Meals were weighed prior to and after consumption. Participants were instructed to “eat until comfortably full” and scored a VAS for palatability of the meal. Energy intake was calculated based on the consumed fraction of the presented meal.

### 2.5 Statistical methods

To estimate the required sample size, a power analysis was performed using a VAS hunger measure as the dependent variable. Estimates of variance components were conducted based on data from a previous study examining the effect of a bitter anorexigenic agent on VAS-based hunger assessments [14]. Power estimates showed 30 participants were sufficient to detect a 10 % difference in the primary outcome measure of VAS hunger, based on a power level of 0.8 and a dropout rate of 20 %. Participant responses collected using VAS were reported as means ± standard error of the means (SEMs). VAS ratings were analyzed with the use of repeated measures linear mixed models, and energy intake at individual meals was analyzed with the use of one-way ANOVA. As per the analysis plan, results are expressed as changes from the baseline (Δ) to reduce possible variation resulting from the prolonged 16 h pre-treatment fast. Tukey's post hoc analysis was used for pairwise comparisons between treatments where main effect ANOVA was significant. Incremental Area Under the Curve (iAUC) for VAS ratings was calculated as the iAUC of the net change (Δ) from baseline measured over 0–510 min with the use of GraphPad Prism software (version 9; GraphPad Software). Statistical significance from placebo treatment was set at *p* ≤ 0.05.

## 3 Results

### 3.1 Participants

Twenty-Seven participants completed all three treatments of the appetite trial. All participants were lean healthy females with a mean age of 21 ± 4 years (range 18–39) and a mean body mass index (BMI) of 21 ± 1 kg/m^2^.

### 3.2 Appetite ratings

**Fig. 3:**
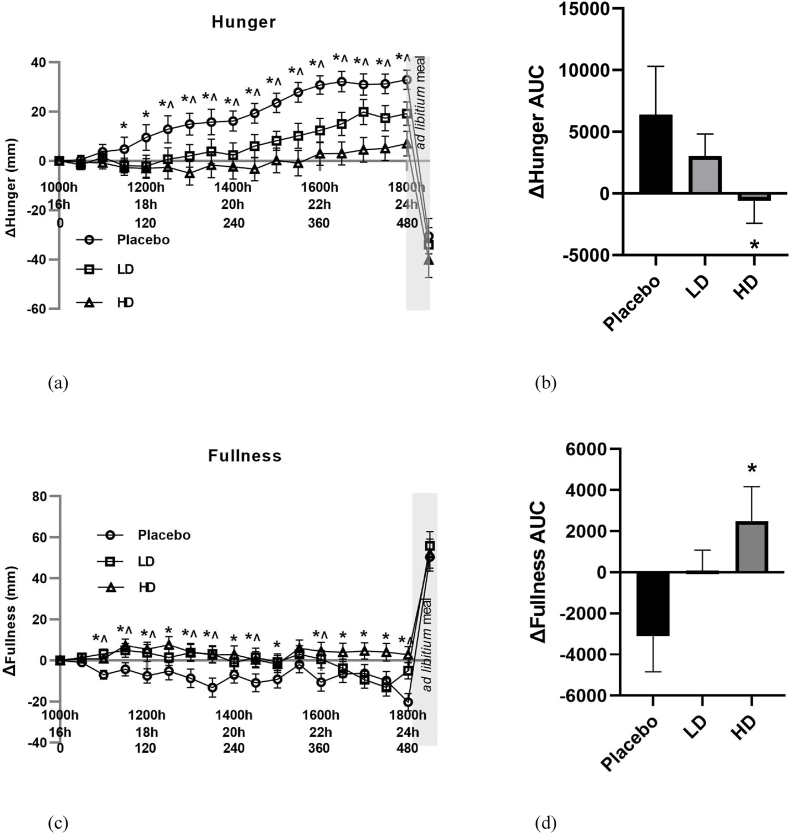
Visual analogue scale (VAS) results for hunger and fullness throughout the day in response to placebo, low dose (LD) and high dose (HD) Amarasate® treatments. Changes (Δ) in the ratings of (a) hunger and (c) fullness. Histograms show AUCΔ0–510 min for Δ with respect to (b) hunger and (d) fullness. Values are means ± standard error of the means (SEMs); n = 27. Treatment capsules were administered at t = 0 min and t = 240 min. Hunger and fullness exhibited a significant main effect for treatment *p* < 0.05 with evidence for a significant treatment × time interaction for hunger *p* ​< ​0.05. Pairwise comparisons: HD vs PLACEBO, ∗*p* < 0.05, LD vs PLACEBO, ˄ *p* < 0.05.

**Fig. 4:**
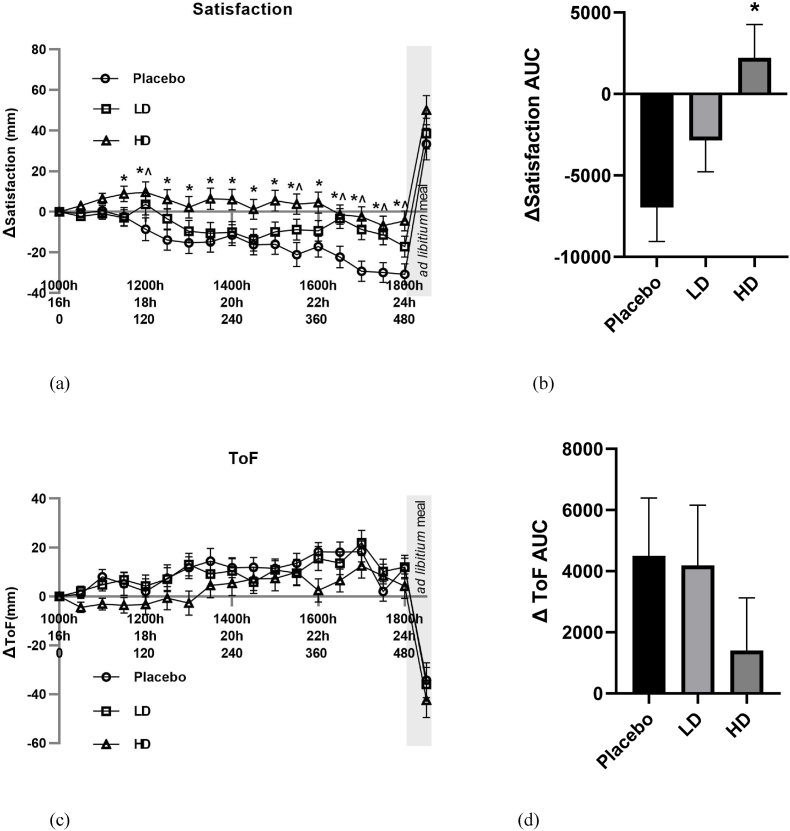
Visual analogue scale (VAS) results for satisfaction and thoughts of food (TOF) throughout the day in response to placebo, low dose (LD) and high dose (HD) Amarasate® treatments. Changes (Δ) in the ratings of (a) satisfaction and (c) TOF. Histograms show AUCΔ0–510 min for Δ with respect to (b) satisfaction and (d) TOF. Values are means ± standard error of the means (SEMs); n = 27. Treatment capsules were administered at t = 0 min and t = 240 min. Satisfaction showed evidence for a main treatment effect (*p* < 0.05) and whereas TOF did not.

The mean change (Δ) in VAS ratings for appetite-related parameters of hunger, TOF, satisfaction and fullness for the three groups are shown in Fig. 3, Fig. 4. The placebo group recorded a 33 mm increase in VAS hunger ratings, a 20 mm decrease in VAS fullness ratings, 12 mm increase in VAS TOF ratings, and a decrease of 31 mm in VAS satisfaction ratings over the 16–24 h fasting period. These changes in appetite measures are as expected and are similar to previous examinations of fasting.

Compared with placebo, both treatments reduced hunger. Mean change (Δ) in VAS hunger ratings was lower compared with the placebo control for both the HD and LD treatment groups. Differences in excess of 10 mm were recorded for Δ hunger for the HD treatment group relative to the placebo for all time points taken from t = 90 min onwards. This same magnitude difference was evident for all time points post t = 150 min for the LD treatment group. These changes in hunger were significant for main treatment effect when examining the entire treatment period (Fig. 3A *p* < 0.05). Post-meal hunger values were −31 mm, −34 mm and −40 mm for the placebo, LD, and HD treatments, respectively, relative to the t = 0 time point. Fig. 3B shows area under the curve (AUC) for Δ hunger over the 510 min period between the start of monitored fasting (t = 0 min) and the final time point (t = 510 min) (ΔAUC). A significant difference was observed between the HD treatment relative to the placebo control (Fig. 4B, *p* < 0.05).

Overall, there was a significant Amarasate treatment effect on fullness (Fig. 3C, *p* < 0.05), as determined by VAS, which showed Δ fullness increases of ≥10 mm relative to the placebo control at time points t = 90, 150, 180, 210, 270, 360, 390, 420, 450, 480 min for the HD treatment, and time points t = 60, 120, 150, 180, 210, 270, 360, 480 for the LD treatment (Fig. 3C). Fig. 3D illustrates the AUC for Δ fullness over the 510 min period between the start of monitored fasting (t = 0 min) and the final time point (t = 510 min) (ΔAUC) and shows a significant difference between the HD and placebo group. Post-meal fullness vales were ​= ​+50 mm, +55 mm, and +52 mm for the placebo, LD and HD, respectively, compared with the t = 0 time point, and show no significant differences between the treatment groups and the placebo.

Recorded VAS-rated changes in satisfaction supported an Amarasate treatment effect (Fig. 4A, *p* < 0.05), there was a 10 mm difference between the HD and placebo for all time points post t = 90 min, and for the LD relative to the placebo at t = 120, 150, 330, 390, 420, 450, 480 min. Post-meal satisfaction values were +33, +39, +50 for the placebo, LD and HD respectively compared with their t ​= ​0 values. Histograms for Δ satisfaction (Fig. 4B) and Δ TOF (Fig. 4D) over the 510 min period between the start of monitored fasting (t = 0 min) and the final time point (t = 510 min) (ΔAUC) showed a significant difference between the HD and placebo treatments for satisfaction only.

VAS ΔTOF ratings did not differ by more than 10 mm between the placebo and LD groups for any of the pre-mealtime points, and at only the 60, 180, 360, and 390 min for the HD group relative to the placebo group. Post-meal TOF ratings of −34 mm, −36 mm, and −42 mm for placebo, LD and HD treatment groups relative to the t = 0 time point, respectively (Fig. 4C).

### 3.3 Craving ratings

**Fig. 5:**
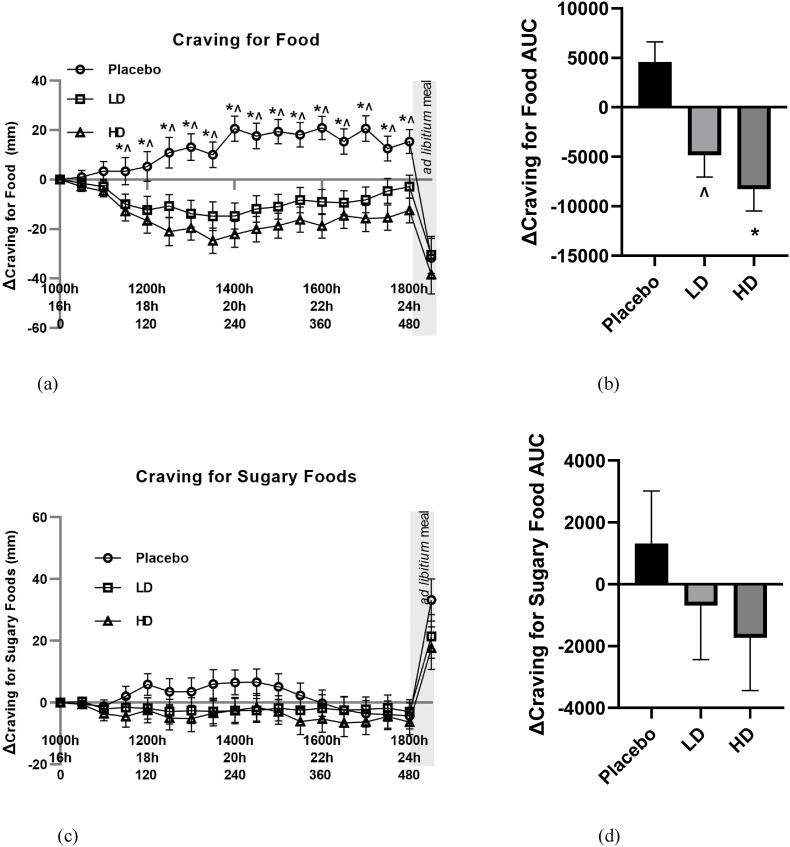
Visual analogue scale (VAS) results for cravings for food and cravings for sugary food throughout the day in response to placebo, low dose (LD) and high dose (HD) Amarasate® treatments. Changes (Δ) in the ratings of (a) cravings for food and (c) cravings for sugary food. Histograms show AUCΔ0–510 min for Δ with respect to (a) cravings for food and (c) cravings for sugary food. Values are means ± standard error of the means (SEMs); n = 27. Treatment capsules were administered at t = 0 min and t = 240 min. Cravings for food exhibited a significant main effect for treatment *p* ​< ​0.05. Pairwise comparisons: HD vs PLACEBO, ∗*p* < 0.05, LD vs PLACEBO, ˄ *p* < 0.05.

**Fig. 6:**
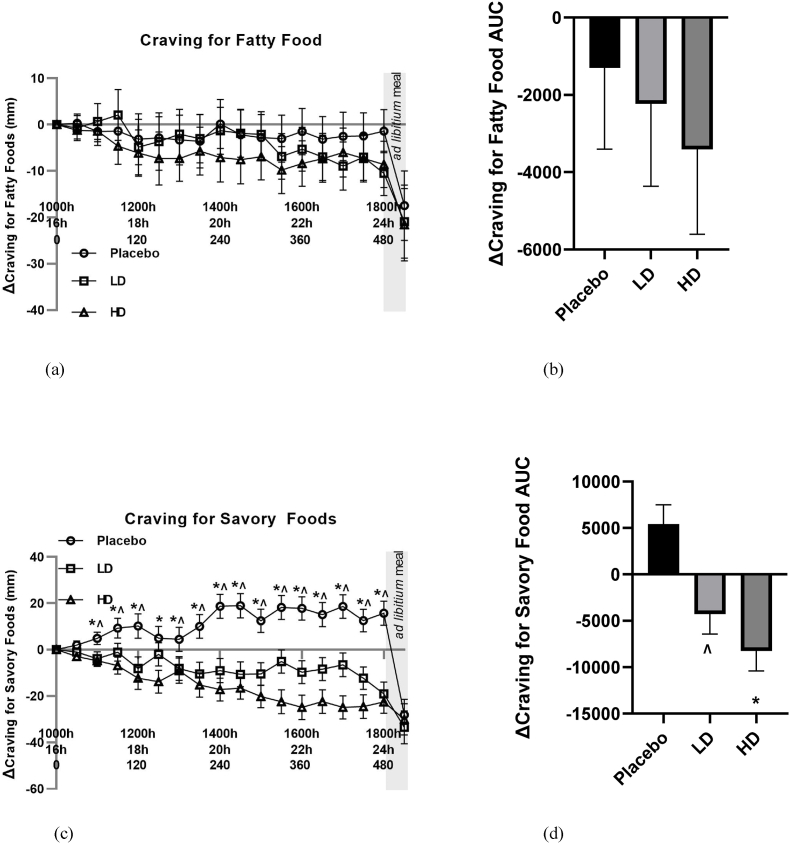
Visual analogue scale (VAS) results for cravings for fatty food and cravings for savory food throughout the day in response to placebo, low dose (LD) and high dose (HD) Amarasate® treatments. Changes (Δ) in the ratings of cravings for fatty food (a) and cravings for savory food (c). Histograms show AUCΔ0–510 min for Δ with respect to cravings for fatty food (a) and cravings for savory food (c). Values are means ± standard error of the means (SEMs); n = 27. Treatment capsules were administered at t = 0 min and t = 240 min. Cravings for savory food exhibited significant main effect for treatment *p* ​< ​0.05. Pairwise comparisons: HD vs PLACEBO, ∗*p* < 0.05, LD vs PLACEBO, ˄ *p* < 0.05.

The mean changes (Δ) in VAS ratings for cravings for “food”, “sugary foods”,” fatty foods”, and “savory foods” are shown in Fig. 5, Fig. 6.

Mean changes (Δ) in VAS food craving ratings were lower compared with the placebo control for both the HD and LD treatments. These changes in hunger were significant for main treatment effect when examining the entire 8 h treatment period (Fig. 5A, *p* < 0.05). Differences in excess of 10 mm were recorded for VAS Δ cravings for food for the HD treatment group relative to the placebo for all time points taken from t = 60 min onwards. This same magnitude of difference was evident for all time points post t = 90 min for the LD treatment group. Post-meal hunger values were −32 mm, −30 mm and −38 mm for the placebo, LD, and HD treatments relative to the t = 0 time point, respectively. Fig. 5B shows the AUC for Δ cravings for food over the 510 min period between the start of monitored fasting (t = 0 min) and the final time point (t = 510 min) (ΔAUC). A significant difference was observed between both the HD treatment and the LD treatment groups relative to the placebo control (Fig. 5B, *p* < 0.05).

Mean changes (Δ) in both VAS sugary food craving and fatty food craving ratings did not differ by 10 mm at any pre-mealtime points between any of the treatments, and at no time point did they differ from their t = 0 baseline by 10 mm (Fig. 5, Fig. 6A). Post-meal sugary food cravings were lower for both the LD and HD treatments relative to the placebo, being +33 mm for the placebo, +21 mm for the LD and +17 for the HD treatments relative to their t ​= ​0 min ​timepoints. Post-meal fatty food cravings were −17 mm for the placebo treatment, 21 mm for the LD treatment and 22 mm for the HD treatment relative to their t = 0 min ​timepoints.

Mean changes (Δ) in VAS Δ cravings for savory food ratings were lower in both the LD and HD treatment groups relative to the placebo treatment with evidence for a main treatment effect (Fig. 6C, *p* < 0.05). Differences in excess of 10 mm were recorded for VAS Δ savory food cravings for all time points post t = 90 for the LD and t = 60 for the HD treatment relative to the placebo treatment. Post-meal VAS savory food craving ratings were −28 mm for the placebo treatment, −33 mm for the LD treatment, and −30 mm for the HD treatment relative to their t = 0 time points. Fig. 6D shows AUC for Δ cravings for savory food over the 510 min period between the start of monitored fasting (t = 0 min) and the final time point (t = 510 min) (ΔAUC). A significant difference was observed between both the HD treatment and the LD treatment groups relative to the placebo control (Fig. 6D, *p* < 0.05).

At no time points did craving for bitter foods, craving for spicy foods, or nausea exceed 10 mm.

### 3.4 Energy intake

Energy intake at the *ad libitum* meal was 14.3 % lower when taking the HD treatment (*p < 0.05)* and 8.1 % lower when taking the LD treatment (not significant) relative to the placebo control treatment.

### 3.5 Side effects and withdrawals

Three participants experienced adverse effects that were deemed likely to be treatment related. Liquid, loose bowel movements were reported by two participants after taking the HD treatment, and from one participant after taking the LD treatment (who also experienced loose stools on the HD treatment), with one participant also experiencing heartburn from the HD treatment. Two additional participants experience adverse effects likely to be study related, but not treatment related. One participant on the placebo treatment experienced nausea and vomited during the fast-breaking meal. One participant withdrew from the study post placebo treatment-day due to post-study day gastrointestinal pain. Additionally, two participants withdrew from the study due to difficulties scheduling study attendance.

## 4 Discussion

This current study shows for the first time that a bitter hop supplement can suppress appetite and can reduce cravings for food in females.

Favorable changes in several appetite measures reported here are likely to be biologically important and to affect eating behavior [13]. These changes in appetite measures occur over a greater than 6 h period, are in excess of the 10 % that is typically targeted for behavioral change, and would be expected to aid fasting compliance [12]. Additionally, the reduced food cravings reported here may also be expected to aid fasting compliance. The results in this study are consistent with those we previously observed in males using a near identical fasting design, with a significant reduction in increased hunger experienced over the last 8 h of the 24 h fasting period compared with placebo. The relative decrease in absolute hunger ratings observed here was a greater magnitude than what was previously seen in males [8]. This finding agrees with other studies showing greater sensitivity of females to the appetite-suppressing effects of GI-targeted bitterness [14]. The greater change in hunger values relative to other appetite measures also agrees with data previously observed in males [8]. Unlike the previous study conducted in males, there was an observed change in how satisfied the participants felt, which may be due to differences in how males and females perceive appetite [15].

It is worth noting that while increases in some appetite and food craving measures were reduced, baseline values were already elevated due to the prior 16 h of fasting and participants would likely be considered to be “hungry” even in the treatment groups. The results presented here indicate a suppression of further hunger, and not a state of no hunger. As observed previously in the males, the increase in hunger around expected lunchtime was blunted in the treatment groups and may be related to inhibition of pro-appetite ghrelin action [16]. Indeed, the same hop extract used in this study has previously been shown to stimulate the release of enteroendocrine cell hormones in people [9], and a potent inhibitor of ghrelin action has recently been identified in enteroendocrine cells [17]. This previous data suggests the inhibition of ghrelin action may have occurred in this study, and that this same inhibition may relate to reduced energy intake at the *ad libitum* fast breaking meal.

Cravings for food in general, and savory food in particular, were seen to be decreased by the bitter hop treatment. Cravings for savory food was the only specific food craving that showed a treatment effect and was very similar in both magnitude and pattern to the changes observed for general food cravings. There were also notable similarities between the hunger and food craving changes observed when on the hop treatments. Interestingly, hunger has previously been correlated with food cravings and suggests that hunger may have influenced the food craving results [18].

The greater increase in cravings for savory foods, when compared to fatty or sugary foods, is in line with a previous report of food cravings experienced during fasting [19]. This savory food craving may also have been driven by participant expectation for the fast-breaking rice-based meal, that would have been considered savory, and it is possible that if a fatty or sugary meal was presented that pre-meal craving profiles and the effect of treatment on them may have been different. It is possible that cravings for savory food was driving the overall food craving, with little apparent influence from either sugary or fatty food craving. Interestingly, post-meal sugar cravings did show a notable absolute difference between treatments. Given that sweet foods are typically consumed as either a between-meal snack or as an after-meal dessert, and that this after meal time point was the only time point where participants experienced significant sugar cravings, it suggests that if a study was conducted that specifically focused on times of heightened sugar cravings, a significant change may be detected.

Amarasate, the bioactive hop extract present in the test capsules, has been shown to exhibit appetite-suppressing activity by increasing the blood concentrations of anorexigenic gut peptides glucagon-like peptide-1 (GLP-1), cholecystokinin (CCK) and peptide tyrosine tyrosine (PYY). These peptide hormones are released during intestinal exposure to bitterness, and in addition to inducing satiation and enhancing postprandial satiety, they may also have a role in reducing food cravings [20,21]. Current preclinical studies support the view that GLP-1 is a target for reward system–related disorders, such as those present during overeating [21]. It is important to note that as hunger and food cravings may be closely related, further work would be needed to determine if any of the observed changes in food cravings reported here are independent of the effect seen on appetite.

The reduction in absolute food intake observed after taking the HD treatment is broadly in line with the 18 % we previously observed in males when given the same daily dose of the same bitter hop extract and is generally in agreement with studies examining the effect of capsule-based GI delivery of bitter compounds on food intake [9,10].

This study has several limitations regarding application for weight management that were made for both practicality and safety reasons. Primary to this is the use of normal weight individuals, limiting extension of these results for weight loss treatments. This decision was made to ensure consistent ability to safely undertake a 24 h fast, and reasonable participant numbers in a pilot trial. Another major limitation is that this study is acute and does not assess any accommodation to the intervention that may occur over longer term use. Finally, this is a relatively small trial, that would benefit from a confirmatory clinical trial with a larger number of study participants. These points should be assessed in future work.

## 5 Conclusions

In conclusion, we determined the efficacy of a bitter hop extract to regulate appetite during the 16–24 h period of a water-only fast and showed that the GI-targeted delivery of a highly bitter hop flower extract can reduce both appetite and food cravings as well as reduce post-fasting-food intake. The results presented here support the effectiveness of this bitter hop extract as an appetite suppressant in females and suggests a role in the suppression of food cravings. This is a growing body of work supporting the concentrated GI bitterness on acute appetite measures, laying the scientific bases for longer-term weight loss studies to be conducted on overweight individuals.

## Author Contributions

Conceptualization, EW, KL and GP; methodology, EW, KL; investigation, EW, KL; data curation, EW, KL; writing—original draft preparation, EW; writing—review and editing, EW, KL and GP; project administration, EW; funding acquisition, EW.

## Funding

This research was funded by Calocurb Limited. The 10.13039/100016205APC was funded by The 10.13039/100011880New Zealand Institute for Plant and Food Research Limited. Calocurb Ltd has had no involvement in the collection, analyses, or interpretation of data; the drafting of the manuscript; or in the decision to publish the results.

## Institutional review Board Statement

Clinical research was approved by the Northern B Health and Disability Ethics Committee (2022 EXP 10995). All studies were carried out in accordance with the Declaration of Helsinki and the trial was registered at the Australian New Zealand Clinical Trials Registry (ACTRN12622000107729).

## Informed consent Statement

Informed consent was obtained from all subjects involved in the study.

## Data Availability Statement

This study contains data that is subject to both ethical and commercial considerations.

## Declaration of Artificial Intelligence (AI) and AI-assisted technologies

During the preparation of this work the author(s) did not use AI.

## Declaration of competing interest

The authors themselves declare no competing financial interests. The New Zealand Institute for Plant and Food Research Limited, a New Zealand government-owned Crown Research Institute, has licensed a hop extract as a dietary supplement to Calocurb Ltd to commercialize and currently holds a minor shareholding in this company.
